# Supplementary material for: Association of FKBP5 polymorphisms with patient susceptibility to coronary artery disease comorbid with depression
Source: PeerJ. 2020 Jun 3;8:e9286. doi: 10.7717/peerj.9286 (PMC7275678; doi:10.7717/peerj.9286)
Supplement: Supplemental Information 1 [file peerj-08-9286-s001.docx]

| SNP | Genotype/  allele | Case,(%) | Control,(%) | *P* value^a^ (χ^2^) | OR (95% CI) | *P* value^b^ |
| --- | --- | --- | --- | --- | --- | --- |
| rs1360780 | CC | 67 (52.3) | 28 (53.9) | 0.479 (1.470) | 1.00 | Referent |
|  | CT | 53 (41.4) | 23 (44.2) |  | 0.963 (0.498-1.861) | 0.911 |
|  | TT | 8 (6.3) | 1 (1.9) |  | 3.342 (0.399-27.997) | 0.266 |
|  | CT+TT | 61 (47.7) | 24 (46.1) | 0.855 (0.033) | 0.659 (0.414-1.048) | 0.855 |
|  | C | 187 (73.0) | 79 (76.0) | 0.568 (0.326) | 1.00 | Referent |
|  | T | 69 (27.0) | 25 (24.0) |  | 1.166 (0.688-1.976) | 0.568 |
| rs2817032 | TT | 71 (55.5) | 30 (57.7) | 0.812 (0.416) | 1.00 | Referent |
|  | TC | 46 (35.9) | 19 (36.5) |  | 1.023 (0.516-2.027) | 0.948 |
|  | CC | 11 (8.6) | 3 (5.8) |  | 1.549 (0.403-5.953) | 0.524 |
|  | TC+CC | 57 (44.5) | 22 (42.3) | 0.785 (0.074) | 1.095 (0.571-2.100) | 0.785 |
|  | T | 188 (73.4) | 79 (76.0) | 0.926 (0.009) | 1.00 | Referent |
|  | C | 58 (26.6) | 25 (24.0) |  | 0.975 (0.570-1.669) | 0.926 |
| rs2817035 | GG | 57 (44.5) | 27 (51.9) | 0.232 (2.925) | 1.00 | Referent |
|  | GA | 65 (50.8) | 25 (48.1) |  | 1.232 (0.643-2.359) | 0.530 |
|  | AA | 6 (4.7) | 0 (0.0) |  | -- | -- |
|  | GA+AA | 71 (55.5) | 25 (48.1) | 0.368 (0.812) | 1.345 (0.705-2.567) | 0.368 |
|  | G | 179 (70.0) | 79 (76.0) | 0.249 (1.329) | 1.00 | Referent |
|  | A | 77 (30.0) | 25 (24.0) |  | 1.359 (0.806-2.294) | 0.250 |
| rs9296158 | GG | 57 (44.5) | 22 (42.3) | 0.799 (0.448) | 1.00 | Referent |
|  | GA | 56 (43.8) | 22 (42.3) |  | 0.982 (0.490-1.972) | 0.960 |
|  | AA | 15 (11.7) | 8 (15.4) |  | 0.724 (0.269-1.946) | 0.522 |
|  | GA+AA | 71 (55.5) | 30 (57.7) | 0.785 (0.074) | 0.913 (0.467-1.752) | 0.785 |
|  | G | 170 (66.4) | 66 (63.5) | 0.594 (0.284) | 1.00 | Referent |
|  | A | 86 (33.6) | 38 (36.5) |  | 0.879 (0.546-1.414) | 0.594 |
| rs9470079 | GG | 61 (47.6) | 19 (36.5) | 0.392 (1.874) | 1.00 | Referent |
|  | GA | 54 (42.2) | 27 (52.0) |  | 0.623 (0.312-1.244) | 0.180 |
|  | AA | 13 (10.2) | 6 (11.5) |  | 0.675 (0.226-2.019) | 0.482 |
|  | GA+AA | 67 (52.4) | 33 (63.5) | 0.174 (1.851) | 0.632 (0.326-1.227) | 0.175 |
|  | G | 176 (68.8) | 65 (62.5) | 0.253 (1.305) | 1.00 | Referent |
|  | A | 80 (31.2) | 39 (37.5) |  | 0.758 (0.470-1.221) | 0.254 |
| rs4713902 | TT | 74 (57.8) | 36 (69.2) | 0.226 (2.971) | 1.00 | Referent |
|  | TC | 49 (38.3) | 13 (25.0) |  | 1.834 (0.884-3.804) | 0.103 |
|  | CC | 5 (3.9) | 3 (5.8) |  | 0.811 (0.184-3.582) | 0.782 |
|  | TC+CC | 54 (42.2) | 16 (30.8) | 0.154 (2.209) | 1.642 (0.827-3.259) | 0.156 |
|  | T | 197 (77.0) | 85 (81.7) | 0.319 (0.995) | 1.00 | Referent |
|  | C | 59 (23.0) | 19 (18.3) |  | 1.340 (0.753-2.384) | 0.320 |
| rs3800373 | CC | 35 (27.3) | 17 (32.7) | 0.727 (0.639) | 1.00 | Referent |
|  | CA | 86 (67.2) | 33 (63.5) |  | 1.700 (0.318-9.075) | 0.535 |
|  | AA | 7 (5.5) | 2 (3.8) |  | 1.266 (0.626-2.561) | 0.512 |
|  | CA+AA | 93 (72.7) | 35 (67.3) | 0.473 (0.515) | 1.291 (0.642-2.593) | 0.474 |
|  | C | 156 (60.9) | 67 (64.4) | 0.537 (0.381) | 1.00 | Referent |
|  | A | 100 (39.1) | 37 (35.6) |  | 1.161 (0.723-1.864) | 0.537 |

Supplementary Table 1. Genotypic and Allelic Distribution of seven FKBP5 Gene Between Male CAD Patients (n=128) and Controls (n =52).

Abbreviations: CI, confidence interval; OR, odds ratio.

^a^*P*value for genotype and allele frequencies in cases and controls using 2-sidedχ^2^ test.
^b^*P*values adjusted by age and gender using logistic regression.

* *P*<0.05

Supplementary Table 2. Genotypic and Allelic Distribution of seven FKBP5 Gene Between Female CAD Patients (n=142) and Controls (n =61).

| SNP | Genotype/  allele | Case,(%) | Control,(%) | *P* value^a^ (χ^2^) | OR (95% CI) | *P* value^b^ |
| --- | --- | --- | --- | --- | --- | --- |
| rs1360780 | CC | 67 (47.2) | 32 (52.5) | 0.799 (0.499) | 1.00 | Referent |
|  | CT | 68 (47.9) | 26 (42.6) |  | 1.249 (0.673-2.317) | 0.480 |
|  | TT | 7 (4.9) | 3 (4.9) |  | 1.114 (0.270-4.595) | 0.881 |
|  | CT+TT | 75 (52.8) | 29 (47.5) | 0.491 (0.475) | 1.235 (0.677-2.253) | 0.491 |
|  | C | 202 (71.1) | 90 (73.8) | 0.587 (0.295) | 1.00 | Referent |
|  | T | 82 (28.9) | 32 (26.2) |  | 1.142 (0.708-1.842) | 0.587 |
| rs2817032 | TT | 71 (50.0) | 39 (64.0) | 0.172 (3.515) | 1.00 | Referent |
|  | TC | 62 (43.7) | 20 (32.8) |  | 1.703 (0.900-3.222) | 0.102 |
|  | CC | 9 (6.3) | 2 (3.3) |  | 2.472 (0.509-12.015) | 0.262 |
|  | TC+CC | 71 (50.0) | 22 (36.0) | 0.068 (3.337) | 1.773 (0.956-3.287) | 0.069 |
|  | T | 204 (71.8) | 98 (80.3) | 0.072 (3.234) | 1.00 | Referent |
|  | C | 80 (28.2) | 24 (16.7) |  | 1.601 (0.956-2.682) | 0.074 |
| rs2817035 | GG | 64 (45.1) | 34 (55.7) | 0.166 (3.596) | 1.00 | Referent |
|  | GA | 73 (51.4) | 27 (44.3) |  | 1.436 (0.783-2.634) | 0.242 |
|  | AA | 5 (3.5) | 0 (0.0) |  | -- | -- |
|  | GA+AA | 78 (54.9) | 27 (44.3) | 0.163 (1.944) | 1.535 (0.839-2.807) | 0.164 |
|  | G | 201 (70.8) | 95 (77.9) | 0.140 (2.174) | 1.00 | Referent |
|  | A | 83 (29.2) | 27 (22.1) |  | 1.453 (0.883-2.391) | 0.142 |
| rs9296158 | GG | 55 (38.7) | 25 (41.0) | 0.754 (0.566) | 1.00 | Referent |
|  | GA | 65 (45.8) | 29 (47.5) |  | 1.019 (0.535-1.940) | 0.955 |
|  | AA | 22 (15.5) | 7 (11.5) |  | 1.429 (0.540-3.780) | 0.473 |
|  | GA+AA | 87 (61.3) | 36 (59.0) | 0.763 (0.091) | 1.098 (0.596-2.025) | 0.763 |
|  | G | 175 (61.6) | 79 (64.8) | 0.550 (0.358) | 1.00 | Referent |
|  | A | 109 (38.4) | 43 (35.2) |  | 1.144 (0.736-1.780) | 0.550 |
| rs9470079 | GG | 85 (59.9) | 23 (37.7) | 0.014 (8.540)* | 1.00 | Referent |
|  | GA | 58 (40.8) | 31 (50.8) |  | 0.419 (0.220-0.799) | 0.008* |
|  | AA | 9 (6.3) | 7 (11.5) |  | 0.348 (0.117-1.035) | 0.058 |
|  | GA+AA | 57 (47.1) | 38 (62.3) | 0.004 (8.412)* | 0.406 (0.219-0.752) | 0.004* |
|  | G | 210 (73.9) | 77 (63.1) | 0.009 (6.804)* | 1.00 | Referent |
|  | A | 66 (26.1) | 45 (36.9) |  | 0.545 (0.344-0.862) | 0.010* |
| rs4713902 | TT | 71 (50.0) | 37 (60.7) | 0.375 (1.963) | 1.00 | Referent |
|  | TC | 60 (42.2) | 20 (32.8) |  | 1.563 (0.822-2.975) | 0.173 |
|  | CC | 11 (7.8) | 4 (6.6) |  | 1.433 (0.427-4.813) | 0.560 |
|  | TC+CC | 71 (50.0) | 24 (39.3) | 0.163 (1.946) | 1.542 (0.838-2.837) | 0.164 |
|  | T | 202 (71.1) | 94 (77.0) | 0.218 (1.151) | 1.00 | Referent |
|  | C | 82 (28.9) | 28 (23.0) |  | 1.363 (0.832-2.333) | 0.219 |
| rs3800373 | CC | 37 (26.1) | 19 (31.1) | 0.559 (1.164) | 1.00 | Referent |
|  | CA | 96 (67.6) | 40 (65.6) |  | 1.232 (0.634-2.396) | 0.538 |
|  | AA | 9 (6.3) | 2 (3.3) |  | 2.311 (0.453-11.782) | 0.314 |
|  | CA+AA | 105 (73.9) | 42 (68.9) | 0.457 (0.554) | 1.284 (0.664-2.481) | 0.457 |
|  | C | 170 (59.9) | 78 (63.9) | 0.440 (0.596) | 1.00 | Referent |
|  | A | 114 (40.1) | 44 (36.1) |  | 1.189 (0.766-1.844) | 0.440 |

Abbreviations: CI, confidence interval; OR, odds ratio.

^a^*P*value for genotype and allele frequencies in cases and controls using 2-sidedχ^2^ test.
^b^*P*values adjusted by age and gender using logistic regression.

* *P*<0.05
